# Attenuation of cancer proliferation by suppression of glypican-1 and its pleiotropic effects in neoplastic behavior

**DOI:** 10.18632/oncotarget.28388

**Published:** 2023-03-21

**Authors:** Fang Cheng, Victor Chérouvrier Hansson, Grigorios Georgolopoulos, Katrin Mani

**Affiliations:** ^1^Department of Experimental Medical Science, Glycobiology Group, Lund University, Biomedical Center A13, Lund, Sweden; ^2^Genevia Technologies Oy, Tampere 33100, Finland

**Keywords:** Glypican-1, TCGA, bladder carcinoma, hepatocellular carcinoma, glioma

## Abstract

Glypicans (GPC1-6) are associated with tumorigenic processes and their involvement in neoplastic behavior has been discussed in different cancer types. Here, a cancer-wide GPC expression study, using clinical cancer patient data in The Cancer Genome Atlas, reveals net upregulation of *GPC1* and *GPC2* in primary solid tumors, whereas *GPC3*, *GPC5* and *GPC6* display lowered expression pattern compared to normal tissues. Focusing on *GPC1*, survival analyses of the clinical cancer patient data reveal statistically significant correlation between high expression of *GPC1* and poor prognosis in 10 particular cancer types i.e., bladder urothelial carcinoma, brain lower grade glioma, liver hepatocellular carcinoma, colon adenocarcinoma, kidney renal clear cell carcinoma, lung adenocarcinoma, mesothelioma, ovarian serous cystadenocarcinoma, uterine corpus endometrial carcinoma and uveal melanoma. *In vitro* studies targeting *GPC1* expression by CRISPR/Cas9 or siRNA or treatment with an anti-GPC1 antibody resulted in attenuation of proliferation of cancer cells from bladder carcinoma, glioma and hepatocellular carcinoma patients (T24, U87 and HepG2 cells). Further, overexpression of *GPC1* exhibited a significant and negative correlation between *GPC1* expression and proliferation of T24 cells. Attempt to reveal the mechanism through which downregulation of *GPC1* leads to attenuation of tumor growth using systematic Ingenuity Pathway Analysis indicate that suppression of *GPC1* results in ECM-mediated inhibition of specific pro-cancer signaling pathways involving TGF-β and p38 MAPK. Identified differential expression and pleiotropic effects of GPCs in specific cancer types emphasize their potential of as novel diagnostic tools and prognostic factors and open doors for future GPC targeted therapy.

## INTRODUCTION

The environment surrounding a tumor contains various components that influence growth and spread of cancer. These components include: proliferating cancer cells, inflammatory cells that have infiltrated the area, structural support provided by the tumor stroma, blood vessels, and various signaling molecules and matrix components that are secreted by the tumor and its surrounding cells. Aberrant growth factor signaling, insensitivity to growth-inhibitory signals, persistent angiogenesis and capability to invade tissues are results of alterations in cell physiology that give rise to malignant behavior. Glypicans (GPCs) are GPI anchored proteins attached to the outer leaflet of the cell membrane and act as co-receptors for different signaling molecules known for regulation of cell growth, motility and differentiation [1]. Six glypican isoforms (GPC1-6) have been identified in human cells. Intense research on the role of individual GPC in specific cancers has revealed new insights in the mechanisms of action and roles in neoplastic behavior [1–3]. Recent data from retrospective and prospective clinical studies point out the therapeutic value of GPCs, as well as their potential as putative biomarkers and prognostic factors in several cancer types [4–6]. An organized evaluation of the impact of each individual GPC in cancer has only been performed for glypican 2 (GPC2) [7].

Glypican 1 (GPC1) is a cell surface proteoglycan substituted with polyanionic heparan sulfate (HS) chains. Studies show that GPC1 play a role in neoplastic behavior by modifying mitogenic signaling pathways exerted by different growth factors, including fibroblast growth factors (FGFs), heparin-binding epidermal growth factor-like growth factor (HB-EGF), hepatocyte growth factor (HGF), bone-morphogenetic protein (BMP), transforming growth factor beta (TGFβ), WNT and insulin-like growth factor (IGF) [8–10]. Further, GPC1 has been shown to undergo recycling and contribute to clearance of oxidative damaged proteins in cancer and neuronal cells [11]. Cell surface GPC1 is internalized from the cell surface and travels to endosomes. In endosomes, the HS chains of GPC1 are cleaved off by a novel copper, nitric oxide and vitamin C (Cu/NO-vitamin C) dependent reaction releasing free polyanionic HS oligosaccharides. The free HS oligosaccharides then form conjugates with oxidized proteins via anhydromannose residues at the reducing terminals by Amadori rearrangement. The putative HS-oxidized protein conjugates are then transported to proteasome (if membrane-attached) or via nucleus to autophagosomes and then lysosome (if luminal) for terminal degradation. The GPC1 core protein travels further to Golgi where it undergoes glycosylation and decoration with new HS chains, then moves to the cell surface prepared for recycling. GPC1 thereby plays a role in clearance of oxidative damaged proteins [11, 12].

Experimental studies link GPC1 to several types of cancers including pancreatic cancer [13], breast cancer [14], glioblastoma [15], esophageal squamous cell carcinoma (ESCC) [16], colorectal cancer [17], mesothelioma [18], prostate cancer [19], hepatocellular carcinoma [20] and cervical cancer [21] as few examples among many published studies. Further, clinical investigations show that high expression of GPC1 is associated with poor prognosis in glioblastoma [22], esophageal squamous cell carcinoma [23] and pancreatic cancers [24]. Moreover, recent clinical studies have identified GPC1 as a novel prognostic biomarker in patients with advanced pancreatic cancer [25] and pancreatic ductal adenocarcinoma [24].

Despite numerous publications, a systematic characterization of the impact of GPCs in cancer progression has not been reported yet. Capitalizing on public gene expression and clinical data available from The Cancer Genome Atlas (TCGA), a cancer genomics program containing molecular characterization of over 20,000 primary cancers and matched normal tissue from 11,000 patients spanning over 33 cancer types, we have systematically investigated differences in gene expression patterns of GPC family in normal and malignant tissues. Employing a series genetic perturbation experiments including knock-out, knock-down and overexpression assays, we have discovered that downregulation of *GPC1* results in attenuation of cell proliferation across different *in vitro* cancer models. Combining these results with systematic identification of differentially expressed genes and pathway analysis between *GPC1*-high and *GPC1*-low across different TCGA cancer types we identify and propose a mechanism where GPC1 interacts with extracellular matrix mediating signal transduction by mitogenic molecules involving TGF-β and p38 MAPK.

## RESULTS

### GPCs display cancer-specific gene expression patterns

**Figure 1:**
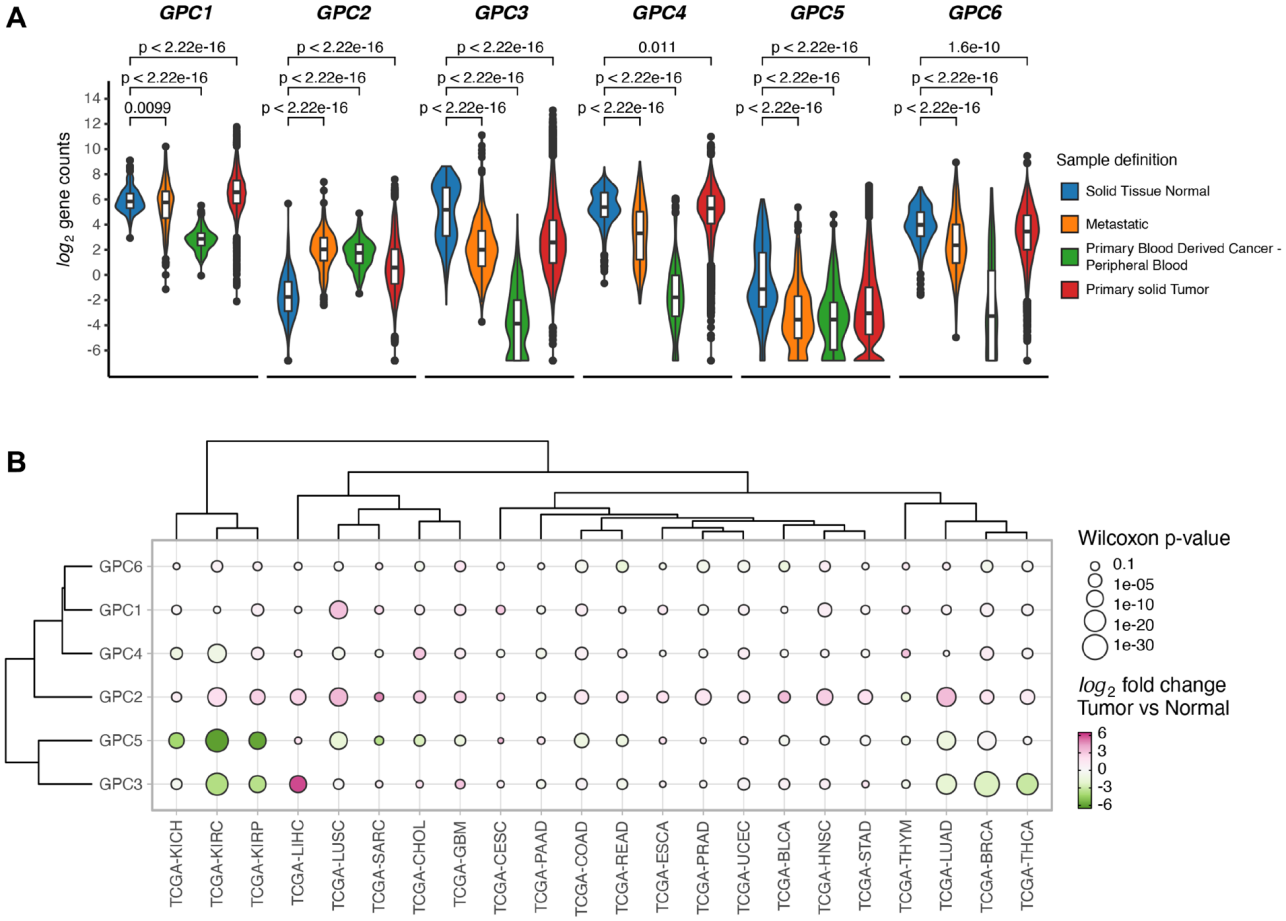
GPCs display sample specific and cancer type specific gene expression differences. (**A**) Difference in expression levels of GPC genes between solid tumors, normal solid tissue, metastatic and blood derived cancers from samples across 33 TCGA projects. Wilcoxon rank sum test *p*-values are reported. (**B**) Clustered bubbleplot of log2 fold changes between tumor and normal samples across 22 cancer types and GPC genes. Points are colored by log2 fold change and the size encodes the -log10 of Wilcoxon rank sum *p*-value. Genes and cancer types are hierarchically clustered based on the Euclidean distance of log2 fold changes.

Expression of each member of GPC family was investigated in primary solid tumors, metastatic tumors and normal solid tissues in TCGA database. Gene expression analysis revealed systematic differences in expression levels between normal and malignant tissues across the GPC gene family (Figure 1A). *GPC1* and *GPC2* displayed significantly elevated expression in primary solid tumor samples compared to healthy tissues while for *GPC3*, *GPC5* and *GPC6* the opposite was true where cancerous tissues were characterized by an overall lower gene expression compared to normal. Beyond the cancer-wide differences, some GPCs exhibited cancer-specific patterns of gene expression. While *GPC1* and *GPC2* exhibited a strong net upregulation in tumor samples over normal across the board of cancer types, other members like *GPC4*, *GPC5*, and *GPC6* were characterized by tissue-specific cancer expression patterns. For example, GPC3 and GPC5 appeared consistently downregulated in kidney tumor samples (TCGA-KIRC, TCGA-KIRP, TCGA-KICH, for abbreviations see Table 1) over normal (Figure 1B). Additionally, GPC3 was also downregulated in breast and thoracic cancer types (TCGA-THYM, TCGA-THCA, TCGA-LUAD, TCGA-BRCA, for abbreviations see Table 1). These findings highlight the pleiotropic effects of gene expression levels of the GPC family in different tumors and their potential as cancer biomarkers.

**Table 1:** List of TCGA projects used in this analysis

Project ID	Description
TCGA-ACC	Adrenocortical carcinoma
TCGA-BLCA	Bladder Urothelial Carcinoma
TCGA-BRCA	Breast invasive carcinoma
TCGA-CESC	Cervical squamous cell carcinoma and endocervical adenocarcinoma
TCGA-CHOL	Cholangiocarcinoma
TCGA-COAD	Colon adenocarcinoma
TCGA-DLBC	Lymphoid Neoplasm Diffuse Large B-cell Lymphoma
TCGA-ESCA	Esophageal carcinoma
TCGA-GBM	Glioblastoma multiforme
TCGA-HNSC	Head and Neck squamous cell carcinoma
TCGA-KICH	Kidney Chromophobe
TCGA-KIRC	Kidney renal clear cell carcinoma
TCGA-KIRP	Kidney renal papillary cell carcinoma
TCGA-LAML	Acute Myeloid Leukemia
TCGA-LGG	Brain Lower Grade Glioma
TCGA-LIHC	Liver hepatocellular carcinoma
TCGA-LUAD	Lung adenocarcinoma
TCGA-LUSC	Lung squamous cell carcinoma
TCGA-MESO	Mesothelioma
TCGA-OV	Ovarian serous cystadenocarcinoma
TCGA-PAAD	Pancreatic adenocarcinoma
TCGA-PCPG	Pheochromocytoma and Paraganglioma
TCGA-PRAD	Prostate adenocarcinoma
TCGA-READ	Rectum adenocarcinoma
TCGA-SARC	Sarcoma
TCGA-SKCM	Skin Cutaneous Melanoma
TCGA-STAD	Stomach adenocarcinoma
TCGA-TGCT	Testicular Germ Cell Tumors
TCGA-THCA	Thyroid carcinoma
TCGA-THYM	Thymoma
TCGA-UCEC	Uterine Corpus Endometrial Carcinoma
TCGA-UCS	Uterine Carcinosarcoma
UVM	Uveal Melanoma

### High expression of *GPC1* is associated with poor survival

**Figure 2:**
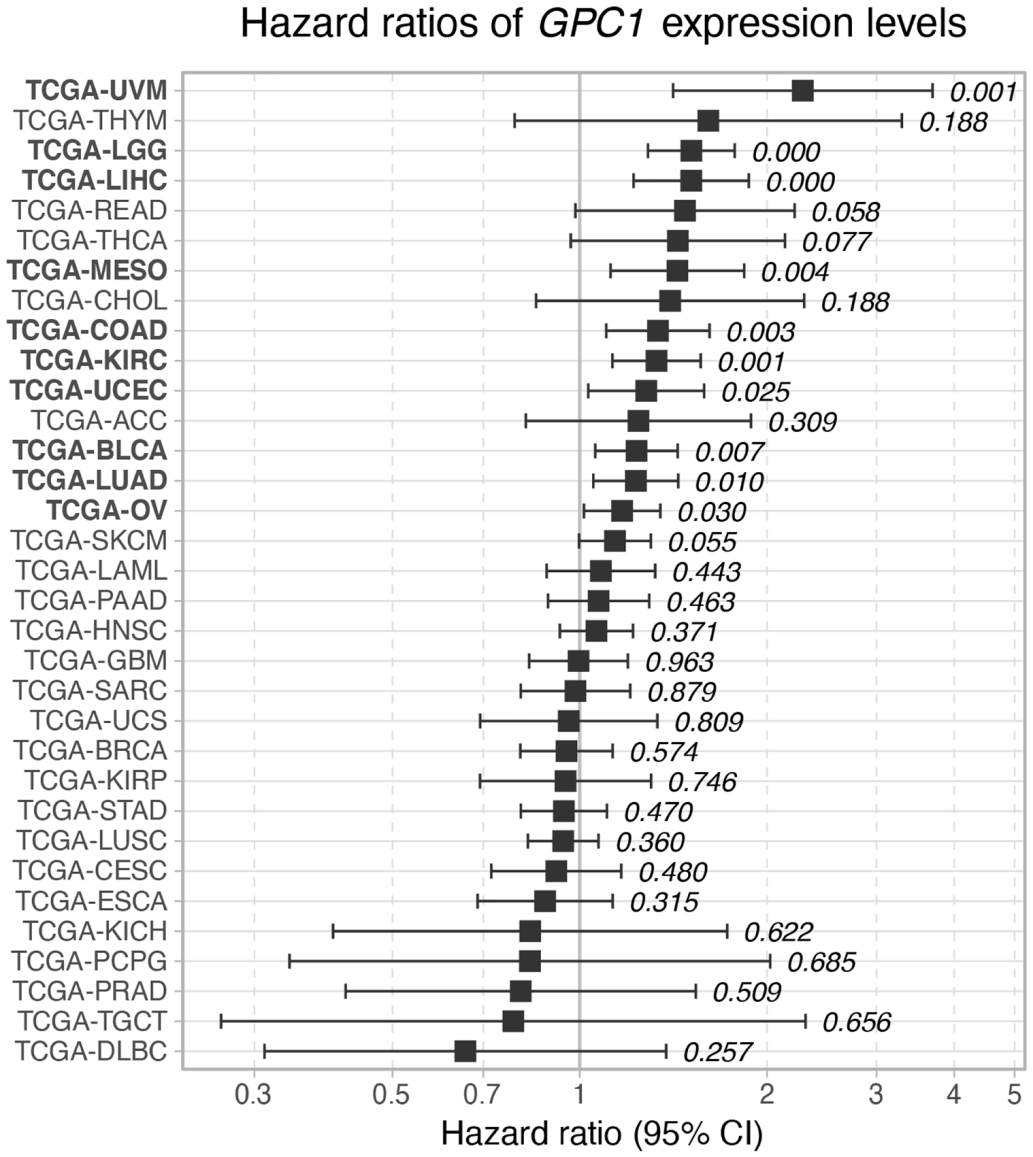
Hazard ratio of *GPC1* expression levels on overall survival across TCGA cancer types. Cox proportional hazard *p*-values are denoted next to each error bar. Error bars represent 95% confidence intervals.

In order to investigate the impact of *GPC1* expression on overall cancer survival, continuous Cox Proportional Hazard (CoxPH) model was fitted against *GPC1* expression values for each TCGA project. The results identified significant negative associations between gene expression levels and survival for 10 cancer types involving bladder urothelial carcinoma (BLCA), colon adenocarcinoma (COAD), kidney renal clear cell carcinoma (KIRC), brain lower grade glioma (LGG), liver hepatocellular carcinoma (LIHC), lung adenocarcinoma (LUAC), mesothelioma (MESO), ovarian serous cystadenocarcinoma (OV), uterine corpus endometrial carcinoma (UCS) and uveal melanoma (UVM) (Figure 2).

We further analyzed the association between *GPC1* expression levels and survival by performing a Kaplan-Meier (KM) survival analysis. Subjects were stratified into 3 bins based on *GPC1* expression (low, medium, and high) (Methods).

**Figure 3:**
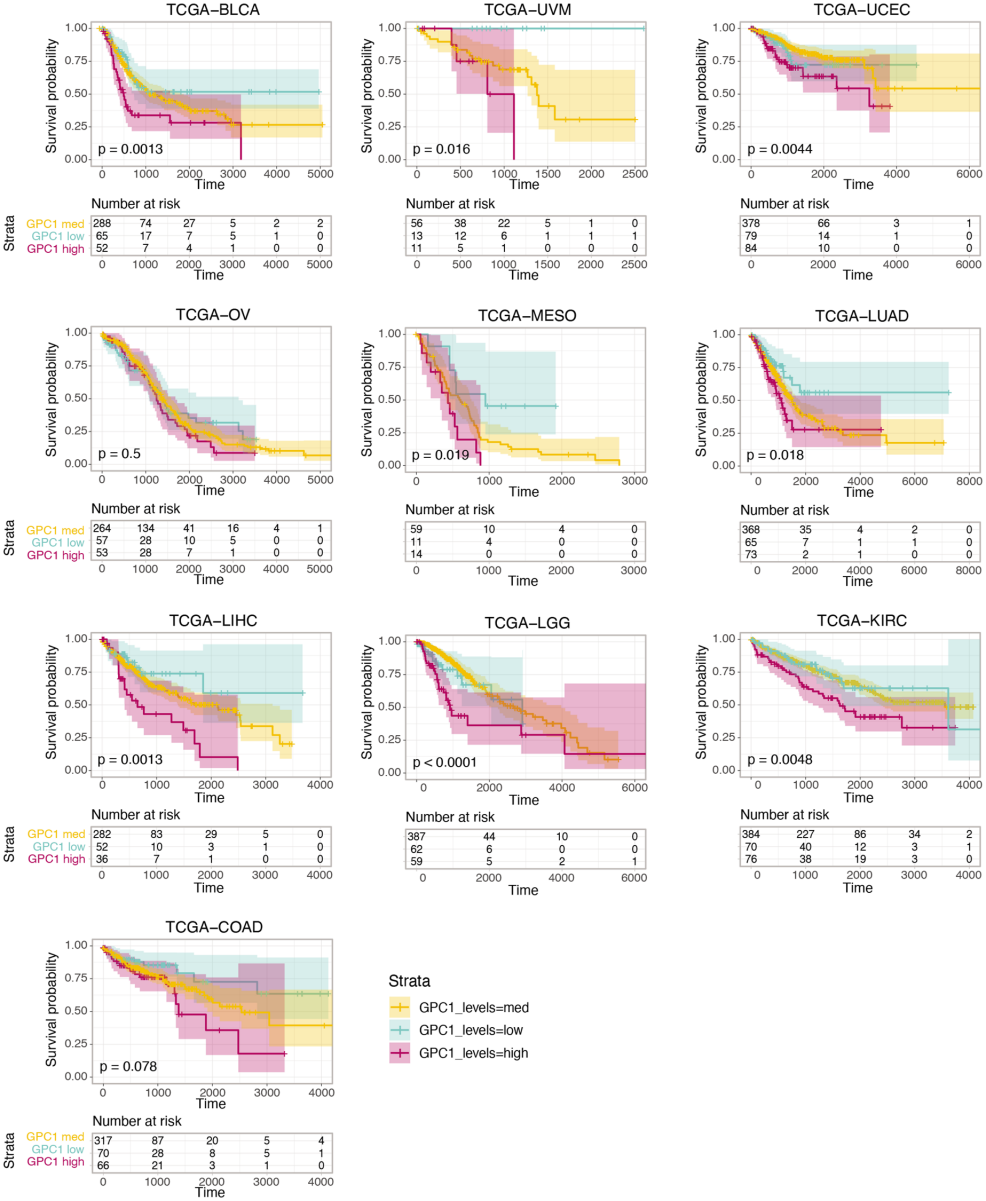
Kaplan-Meier curves of the survival probability of *GPC1* expression strata in indicated TCGA projects where higher expression level is associated with poor prognosis in these particular cancer types. The risk table presents the number of subjects at risk at each time point. Time is represented in days.

As shown in Figure 3, KM curves of the survival probability of GPC1 expression strata in the 10 chosen TCGA projects indicate a statistically significant correlation between higher expression level of GPC1 and poor prognosis in all these 10 specific cancer types.

### Silencing *GPC1* expression attenuates proliferation of cancer cells from urinary bladder carcinoma, brain glioma and liver hepatocellular carcinoma patients

**Figure 4:**
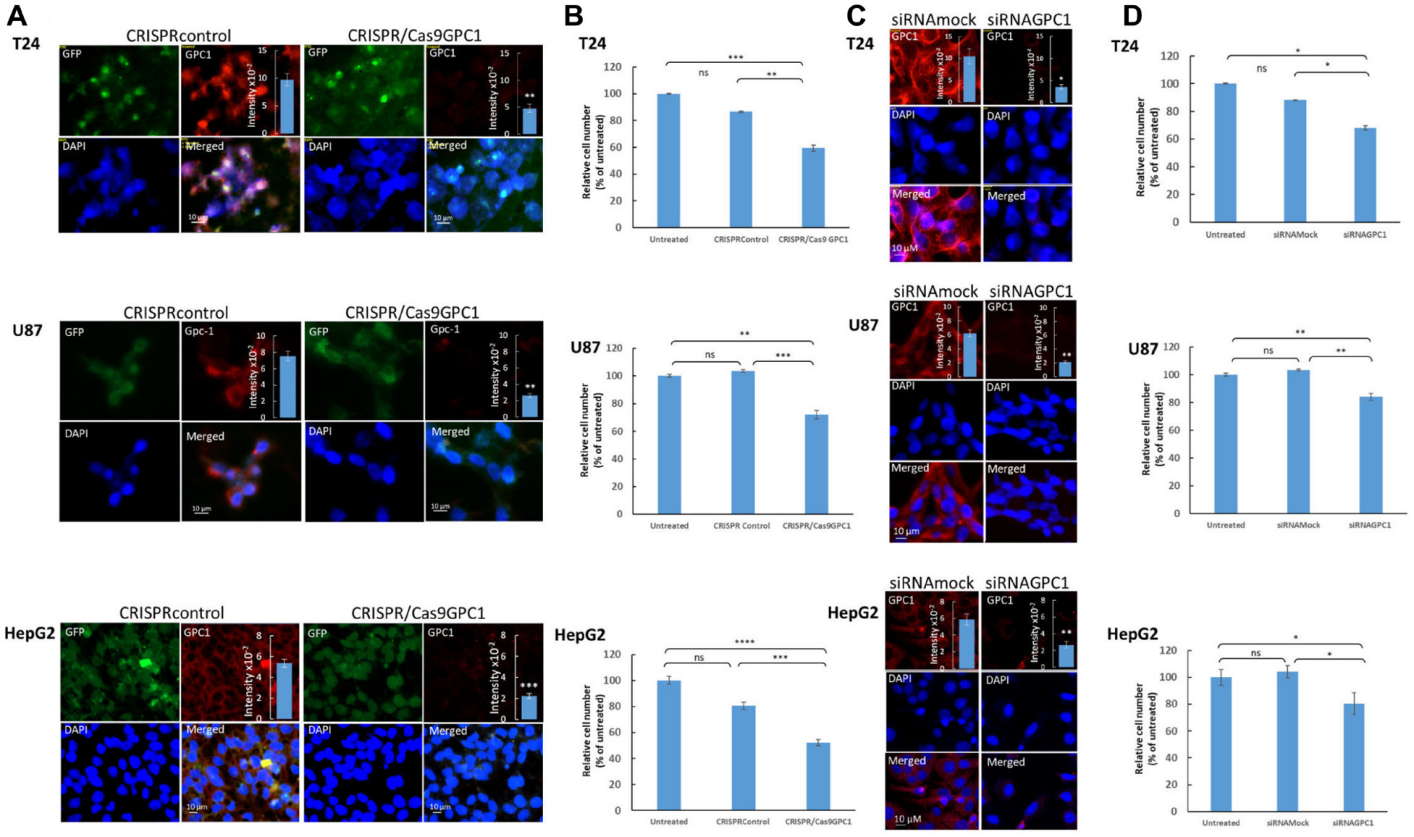
Suppression of *GPC1* expression attenuates proliferation of T24, U87 and HepG2 cells. (**A** and **B**) Depletion of *GPC1* by CRISPR/Cas 9 and (**C** and **D**) by siRNA. (A and C) Depletion of endogenous *GPC1* expression by CRISPR/Cas9 or siRNA, as measured by immunofluorescence microscopy and slot blot assays. T24, U87 and HepG2 cells were transfected with either (A) a CRISPR/Cas9 non-specific construct (not targeting any known gene; CRISPRcontrol) or a CRISPR/Cas9 construct targeting GPC1 (CRISPR/Cas9GPC1) or (C) siRNAGPC1 or siRNAmock (scrambled vector) as indicated in the images. The CRISPR constructs encoded a GFP reporter to indicate transfection. Cells were subsequently fixed in acetone and stained for GPC1 using anti-GPC1 primary antibody and Alexa Fluor 594-tagged goat anti-rabbit IgG. Expression of CRISPR-Cas9 constructs (GFP) and silencing of *GPC1* (Alexa Fluor 594) was monitored by fluorescence microscopy. Counterstaining with DAPI was used to visualize the nuclei (blue). Representative cells for all experiments are shown. Exposure times were the same in all cases. Bar, 10 μm. Insets in (A) and (C), cell extracts containing the same amounts of protein were blotted onto PVDF membranes and probed against anti-GPC1 primary antibody followed by horseradish peroxidase-conjugated anti-rabbit IgG. Loading consistencies were controlled and adjusted after probing with β-tubulin using anti-β-tubulin primary antibody followed by horseradish peroxidase-conjugated anti-mouse IgG. GPC1 signals in the blots were quantified by densitometry. The amount of immuno-reactive GPC1 was significantly lowered in the GPC1 depleted cells (CRISPR/Cas9GPC1 and siRNAGPC1 transfected cells) compared to controls (Student’s *t*-test, two-tailed unequal variances, *N* = 5, ^*^ *P* ≤ 0.05, ^**^ *P* ≤ 0.01 and ^***^ *P* ≤ 0.001). Values shown are means ± SE. (B and D) Effect of depletion of GPC1 expression on proliferation of T24, U87 and HepG2 cells. T24, U87 and HepG2 cells were transfected with either (B) CRISPRcontrol or CRISPR/Cas9GPC1 vector or (D) siRNAmock or siRNAGPC1 vector as indicated in the images. The cell density was determined after 3 days of proliferation. Untreated cells containing only medium were included as controls. The relative cell number was calculated as % of untreated cells. The results are presented in graphs for experiments performed in duplicates, *n* = 5 in each experiment. The data points are shown as the means ± SE. Proliferation of T24, U87 and HepG2 cells was significantly lowered in the CRISPR/Cas9GPC1 and siRNAGPC1 transfected cells compared to the control cells (Student’s *t*-test, two-tailed unequal variances, *N* = 5). Error probabilities of *P* ≤ 0.05 were considered statistically significant. Indication of *P*-values: ns *P* > 0.05, ^*^ *P* ≤ 0.05, ^**^ *P* ≤ 0.01, ^***^ *P* ≤ 0.001 and *P* ≤ 0.0001.

Survival analysis results revealed an association between high level of *GPC1* expression and poor prognosis in several cancer types including BLCA, LGG, and LIHC. To further elucidate the impact of *GPC1* expression on progression of these cancers, the effect of GPC1 repression on proliferation rate in different cancer cell lines was investigated. Specifically, we knocked-out exon 1 of GPC1 with CRISPR/Cas9GPC1 and transiently interfered with GPC1 transcript using siRNAGPC1 in cancer cells from patients with urinary bladder carcinoma (T24 cells), malignant brain glioma (U87 cells) and liver hepatocellular carcinoma (HepG2 cells). Immunofluorescence microscopy indicated substantial decrease of GPC1 protein levels (Figure 4A) and slot blot assays displayed significant decrease of immunereactivity with GPC1 in CRISPR/Cas9GPC1 transfected cells compared to CRISPRcontrols, though the signal was not totally abolished (Figure 4A and insets). Also, knock-down of GPC1 using siRNAGPC1 resulted in a considerable and significant decrease of *GPC1* expression compared to cells transfected with scrambled vectors (siRNAmock) (Figure 4C). Subsequently, we investigated the effect of repression of *GPC1* expression on the proliferation rate of T24, U87 and HepG2 cells. CRISPR/Cas9GPC1 or siRNAGPC1 transfected cells were cultured for 3–4 days and the effect on cell proliferation was compared to cells transfected with CRISPRcontrol or siRNAmock vectors as well as to untreated cells. Targeting *GPC1* with CRISPR/Cas9GPC1 attenuated proliferation of T24, U87 and HepG2 cells significantly (Student’s *t*-test, two-tailed unequal variances, *N* = 5, *P* ≤ 0.01) to 59%, 72% and 52% respectively, compared to untreated controls (Figure 4B). Similarly, siRNA-mediated knock-down of GPC1 resulted in a significant decrease of proliferation rate in all studied cell types compared to siRNAmock (Figure 4D). Taken together these results indicate that suppression of *GPC1* expression both with CRISPR/Cas9GPC1 and siRNAGPC1 attenuates progression of T24, U87 and HepG2 cancers by inhibiting cell proliferation.

**Figure 5:**
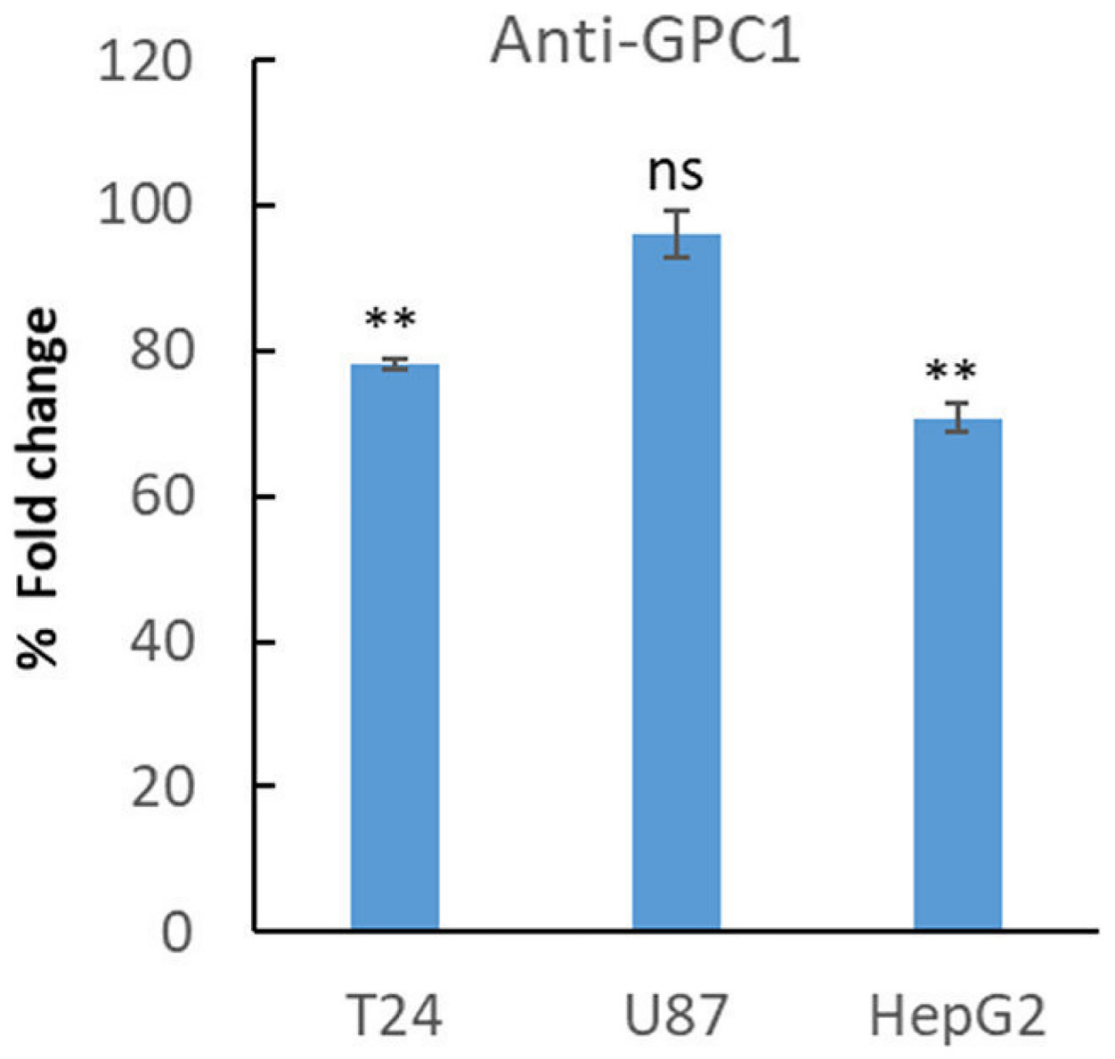
Treatment with anti-GPC1 antibody lowered proliferation of T24 and HepG2 cells. T24, U87 and HepG2 cell were plated at a density of 5 000 cells/well. After 24 h of plating, the cells were left untreated or were treated with an anti-GPC1 polyclonal antibody (1:200). After 3 days the cells were fixed in 0.25% (v/v) glutaraldehyde in Hanks’ balanced salt solution, and the cell density was determined by staining of the nuclei with crystal violet. The relative cell number was calculated as % of fold changes compared to untreated cells. The results are presented in graphs for experiments performed in duplicates, *n* = 5 in each experiment. The data points are shown as the means ± SE. Proliferation of T24 and HepG2 cells was significantly lowered upon treatment with GPC1-antibody compared to untreated cells (Student’s *t*-test, two-tailed unequal variances, *N* = 5). Error probabilities of *P* ≤ 0.05 were considered statistically significant. Indication of *P*-values: ns *P* > 0.05 and ^**^ *P* ≤ 0.01.

In another set of experiments, T24, U87 and HepG2 cells were allowed to proliferate in the presence of a polyclonal antibody against GPC1 for 4 days. Interestingly, treatment with GPC1 antibody reduced proliferation of T24 cells and HepG2 cells significantly (Student’s *t*-test, two-tailed unequal variances, *N* = 5, *P* ≤ 0.01) to ~75% compared to untreated cells, while no effect on proliferation of U87 cells was observed (Figure 5).

### Overexpression of GPC1 augmented proliferation of T24 cells

**Figure 6:**
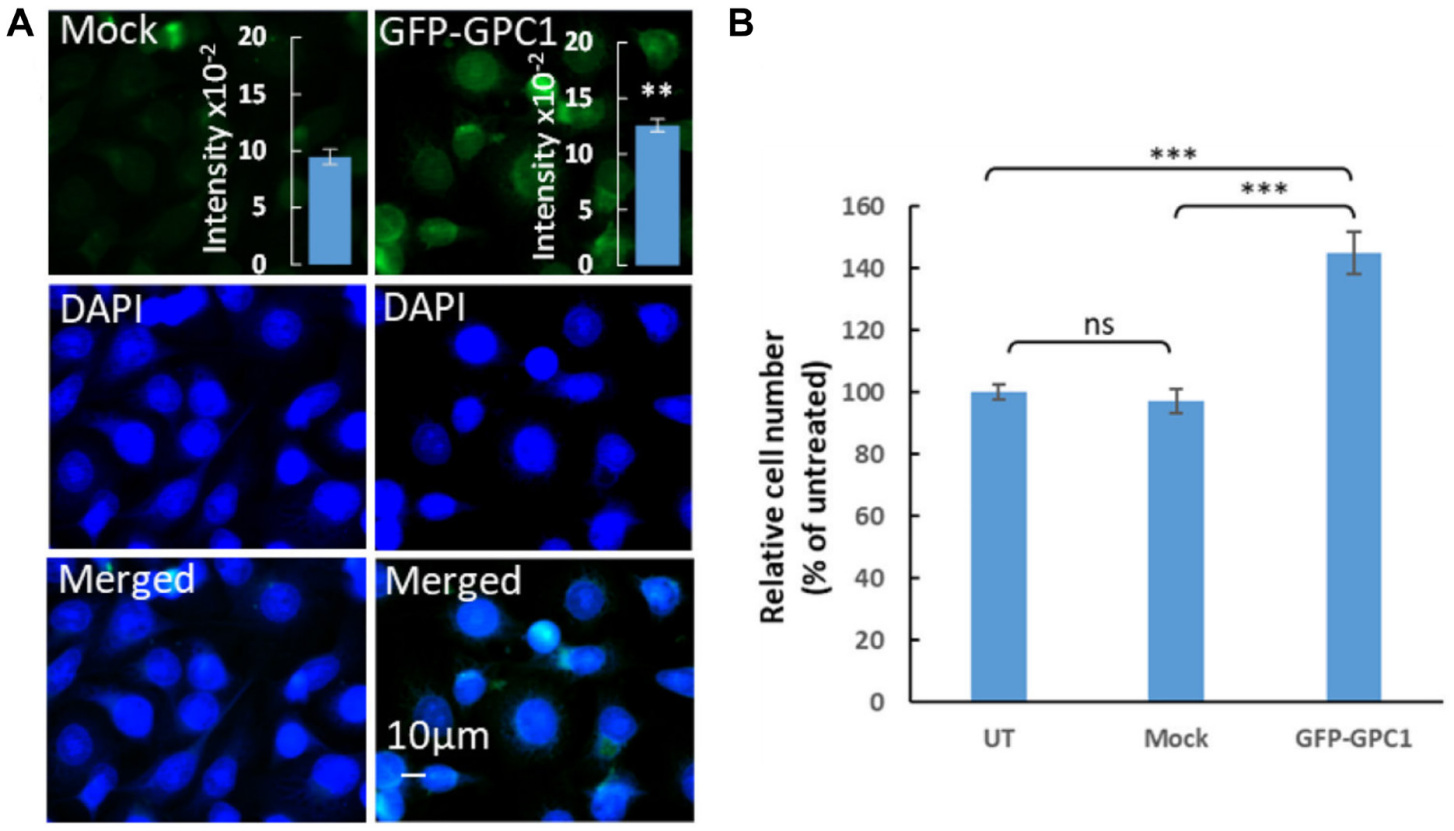
Overexpression of *GPC1* increases proliferation of T24 cells. (**A**) Overexpression of *GPC1* as measured by immunofluorescence microscopy and slot blot assays. T24 cells were transfected with no vector (Mock) or a *GPC1* overexpression vector containing a GFPSpark (GFP-GPC1) as indicated in the images. After fixation with acetone and counterstaining with DAPI (to visualized the cells nuclei, blue), the overexpression of *GPC1* was detected by fluorescence microscopy (GFP-GPC1). Representative cells are shown for Mock and GFP-GPC1. Exposure times were the same in all cases. Bar, 10 μm. Insets in (A), cell extracts containing the same amounts of protein were blotted onto PVDF membranes and probed using anti-GPC1 primary antibody followed by horseradish peroxidase-conjugated anti-rabbit IgG. Loading consistencies were controlled and adjusted after probing with β-tubulin using anti-β-tubulin primary antibody followed by horseradish peroxidase-conjugated anti-mouse IgG. GPC1 signals in the blots were quantified by densitometry. The amount of immuno-reactive GPC1 was significantly increased in GFP-GPC1 transfected cells compared to the Mock (Student’s *t*-test, two-tailed unequal variances, *N* = 5, ^**^ *P* ≤ 0.01). Values shown are means ± SE. (**B**) Effect of overexpression of *GPC1* on proliferation of T24. T24 cells were transfected with GFP-GPC1 or Mock (no vector). Untreated cells containing only medium were included as control. The cell density was determined after 4 days of proliferation. The relative cell number was calculated as % of untreated cells. The results are presented in graphs where *n* = 5. The data points are shown as the means ± SE. Proliferation of T24 cells significantly increased in the GFP-GPC1 transfected cells compared to the Mock (Student’s *t*-test, two-tailed unequal variances, *N* = 5). Error probabilities of *P* ≤ 0.05 were considered statistically significant. Indication of *P*-value summaries: ns *P* > 0.05 and ^***^ *P* ≤ 0.001.

Effect of GPC1 on proliferation of T24 cells was also studied using overexpression experiments. In these experiments T24 cells were transfected with a GPC1 overexpression vector (GFP-GPC1) and the effect on the proliferation rate was investigated. Since the GPC1 overexpression vector contained a GFPSpark, expression of the vector was monitored by tracing GFP using fluoresce microscopy (Figure 6A). For quantitative analysis, overexpression of GPC1 was investigated in extracts of T24 cells transfected with GFP-GPC1 or Mock (omitting the GFP-GPC1 vector) by slot blot assays using anti-GPC1 antibody (insets in Figure 6A). Immunofluorescence microscopy showed an overexpression of GPC1 and slot blot assays confirmed a significant increase in immunoreactivity with GPC1 antibody in GFP-GPC1 transfected cells compared to Mock (Student’s *t*-test, two-tailed unequal variances, *N* = 5, ^**^ *P* ≤ 0.01) (Figure 6A). In proliferation studies overexpression of GPC1 resulted in significantly increased proliferation rate of T24 cells to 140% compared to untreated or Mock cells (Student’s *t*-test, two-tailed unequal variances, *N* = 5, ^***^ *P* ≤ 0.001) (Figure 6B). This was in line with the clinical TCGA data from bladder urothelial carcinoma (BLCA) patients where a CoxPH models and KM survival analysis revealed poor survival associated with high expression of *GPC1* (see Figures 2 and 3 TCGA-BLCA).

### Suppression of GPC1 results in ECM-mediated inhibition of pro-cancer and proliferation signaling pathways

**Figure 7:**
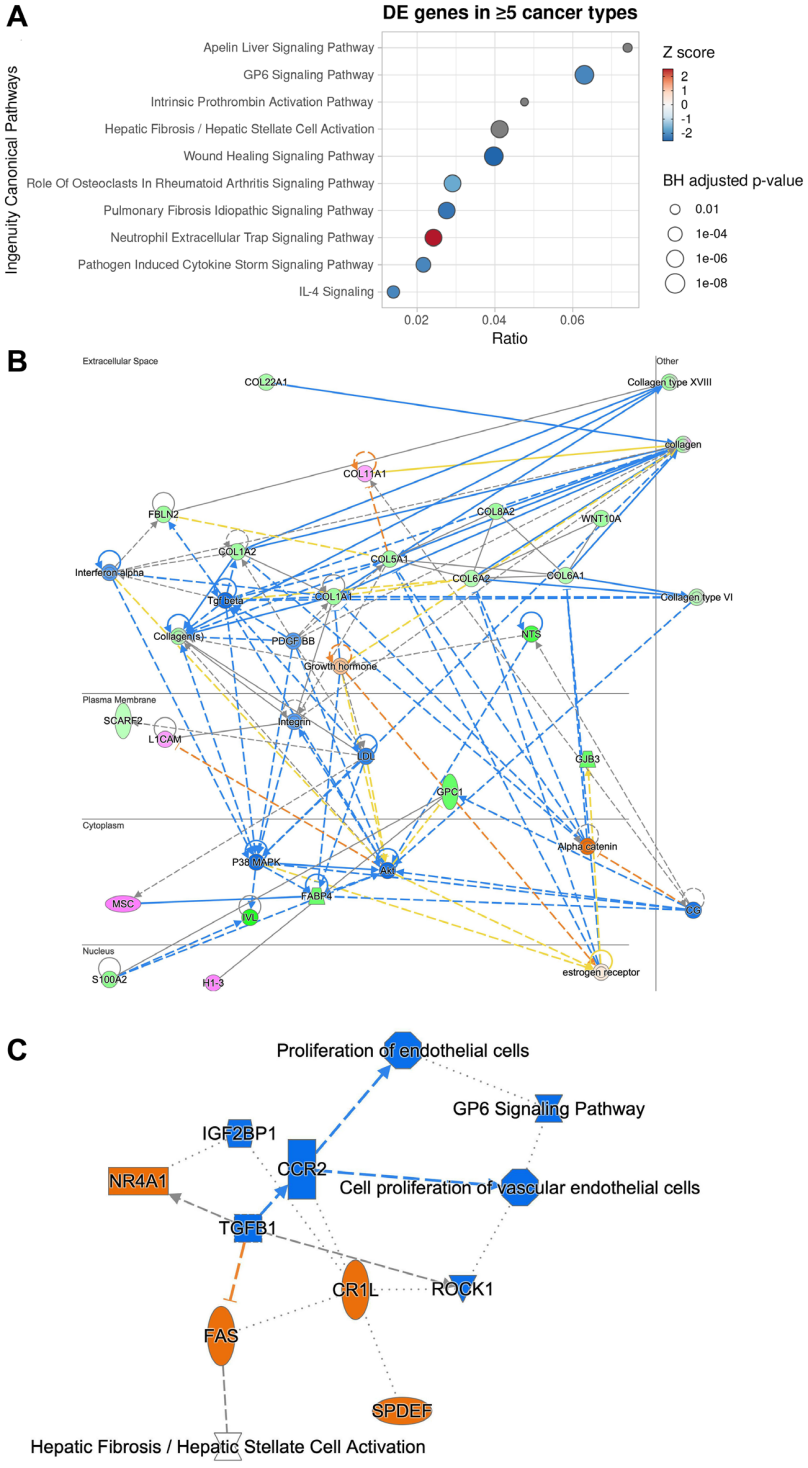
Ingenuity pathway analysis of 66 genes differentially expressed in at least 5 cancer types between *GPC1*-high and *GPC1*-low cancer patients reveals potential mechanism through which GPC1 modulates cell proliferation. (**A**) Dotplot of the top 10 canonical pathways enriched by enrichment ratio (x-axis). Predicted activity score (z-score) is color-coded where z-score >0 denotes activation while z-score < 0 denotes inhibition. Pathways with no or near-zero prediction are grey-colored. Size of the dots denotes the Benjamini-Hochberg adjusted *p*-value (−log10). (**B**) Network graph of the most significant IPA-generated network with subcellular localization layout. Network nodes are colored by observed gene expression fold changes (pink: upregulation; green: downregulation). (**C**) Graphical summary of Molecule Activity Prediction (MAP) analysis with predicted down-stream and up-stream activation or inhibition of molecules and/or processes based on the observed expression changes (orange: predicted activation; blue: predicted inhibition).

To gain insights into potential mechanism through which downregulation of GPC1 gene leads to attenuation of proliferation we performed systematic differential expression (DE) analysis between *GPC1*-low and *GPC1*-high subjects across different TCGA cancer type datasets followed by Ingenuity Pathway Analysis (IPA). For this analysis we considered 10 TCGA cancer types with significant CoxPH results across *GPC1* expression levels. Genes that were identified as differentially expressed (Benjamini-Hochberg adjusted *p*-value <0.05 and absolute log2 Fold Change >0.58) between *GPC1*-low and *GPC1*-high patient groups in at least 5 of the studied TCGA cancer types (66 total genes) were then subjected to IPA core analysis (Methods). Pathway analysis revealed that genes deregulated between *GPC1*-low and *GPC1*-high patients are associated with pathways involved in immunological aspects of cancer progression and metastatic dissemination including activation of neutrophil extracellular trap signaling pathway and inhibition of pathogen induced cytokine storm signaling pathway as well as IL-4 signaling (Figure 7A). Of note, IL-4 is often associated with tumor where among its biological functions can promote proliferation and survival of cancer cells and its autocrine origin is an indicator of tumor aggressiveness [26]. Additional enriched terms include fibrosis and wound healing, both mechanisms whose deregulation can lead to cancer [27, 28]. Upon inspection of genes overlapping with these terms we find genes of the collagen family across the significant terms (Supplementary Table 1) suggestive of mechanisms involving extracellular matrix (ECM) organization, adhesion, cancer metastasis and invasion [1, 29]. Notably, collagen deregulation and ECM destabilization is a mechanism common in cancer fibrosis [29] and GPC1 has been shown to directly interact with collagen in breast tumor growth regulation [30].

Network reconstruction of the predicted relationships between molecules as inferred from gene expression changes reveal that *GPC1*-low patients reveals inhibition of linchpin regulators positively associated with cancer maintenance and progression, including transforming growth factor beta 1 (TGFβ), p38 MAPK, AKT, and PDGF-BB, interferon-α and WNT [For review see 1, 9, 10, 31, 32] (Figure 7B). Moreover, direct interaction was predicted between GPC1 and S100A2 which encodes A2 member of the S100 proteins family, highly involved in regulation of cell cycle and differentiation via its 2 EF-hand calcium-binding motif [33]. Also, direct interaction was predicted between GPC1 and H1-3 which encodes H1.3 linker histone indicating involvement of chromatin remodeling, nucleosome spacing and DNA methylation and thereby regulation of gene transcription (Figure 7B).

Molecule Activity Prediction (MAP) analysis predicted inhibition of TGFB1 cytokine as a central node, suggesting interference with mitogenic pathways that involve TGFB receptor signaling in cancer, i.e., cell proliferation and epithelial-to-mesenchymal transition (EMT) [9], a core process of fibrosis and wound healing [34]. Further, inhibition of TGFB1 was predicted to be connected with downstream inactivation of gene for C-C motif chemokine receptor 2 (CCR2) and interference with proliferation of vascular endothelial cells and angiogenesis. Furthermore, suppression of TGFB1 was predicted to result in activation of genes for nuclear receptor subfamily 4 group A member 1 (NR4A1) and FAS receptor suggesting interference with processes involving apoptosis (Figure 7C). Taken together, a potential mechanism of proliferation attenuation through *GPC1* repression involve negative interaction with ECM molecules (collagen family) [10] resulting in reduced mobilization of pro-cancer and pro-inflammatory molecules from the extracellular space such as TGFβ and IFNα which in turn result in reduced P38 MAPK activation, one of the key regulators of normal and malignant cell proliferation [35].

## DISCUSSION

Deciphering the mechanisms involved in communication between cancer cells and with stroma is crucial for finding new biomarkers, prognostic factors and therapeutic strategies. GPCs are located at the cell surface and cooperate with signaling molecules that control growth, movement, and differentiation. It is, therefore, not surprising that implication of GPCs in tumorigenesis and cancer therapy has been subjected to numerous investigations [36, 37]. Growing body of evidence indicate involvement of members of GPC family in cancer development, however, an organized and cancer-wide investigation of the impact of GPCs on cancer progression has never been performed. Here, a systematic gene expression analysis using clinical patient data in the TCGA database revealed that GPCs undergo general alterations in cancer where *GPC1* and *GPC2* exhibit significantly higher expression levels in primary solid tumors whereas *GPC3*, *GPC5* and *GPC6* display an overall lower expression levels compared to normal tissues. Also, specific members of GPC family showed to exhibit an anatomical and organ-specific cancer association. For example, expression of *GPC5* showed to be suppressed in different kidney cancers and expression of *GPC3* appeared to be downregulated in several thoracic associated cancers including breast cancer. Additional analysis unveiled those certain members of GPC family display expression alterations associated with specific cancer types. For instance, comparison between normal liver and kidney tissue and cancer involving these organs revealed high expression of *GPC3* in liver hepatocellular carcinoma (LIHC) and low expression of *GPC5* in kidney renal clear cell carcinoma (KIRC). These findings point out the pleiotropic effects of expression of GPCs in different cancers.

Focusing on GPC1, continuous CoxPH analysis, which allows description of survival time as a function of prognostic factor and diagnostic tool, revealed statistically significant and negative correlation between *GPC1* expression levels and patients’ survival in 10 particular cancer types i.e., aggressive carcinomas in bladder, colon, kidney, liver, lung, ovary and uterus (BLCA, COAD, KIRC, LIHC, LUAC, OV and UCS) as well as in glioma (LGG), mesothelioma (MESO) and uveal melanoma (UVM). Further investigations by KM survival analysis of *GPC1* expression strata in these cancers unveiled correlation between high expression of GPC1 and poor prognosis pointing out the value of GPC1 as diagnostic tool or prognostic factor in these cancers. *In vitro* investigations by overexpression of GPC1 protein in T24 bladder carcinoma cells resulted in augmented proliferation. In contrary, repression of GPC1 gene expression in T24 bladder carcinoma, HepG2 liver hepatocellular carcinoma and U87 brain glioma cells using CRSPR/Cas9 or siRNA resulted in significant attenuation of cancer cell proliferation indicating the potential of GPC1 as target for future cancer therapies.

Immunodiagnostic and immunotherapy are emerging approaches in detection and treatment of cancers. Here, treatment with an in house made GPC1 antibody decreased proliferation of T24 bladder carcinoma cells and HepG2 liver hepatocellular carcinoma cells. However, U87 glioma cells were not sensitive to this antibody. It is well known that depending on the epitope, there can be variations in consistencies between RNA levels and antibodies’ recognition pattern. As example, data from The Human Protein Atlas shows difference in detection levels and expression patterns for two different GPC1 antibodies in biopsy samples from malignant glioma patients (Supplementary Figure 1A and 1B; https://www.proteinatlas.org/). Also, variations within detection levels for the same antibody in the same cancer type can occur emphasizing the importance of precision and personalized form of therapy (Supplementary Figure 1C). Recent studies in animal models and clinical studies in cancer patients resemble potential of using anti-GPC1 antibody for detection of bladder cancer using fluorescence imaging [38] or utilizing GPC-1 directed radioimmunotherapy in different solid tumors [39]. Further, GPC1 targeted positron emission tomography (PET) was recently tested with positive results as a novel diagnostic and therapeutic tool in glioblastoma and prostate cancer [40, 41].

Evidence has shown that the specific function of individual proteoglycans such as GPCs depends on the structure of the core protein and their HS chains [1]. Due to their cell surface localization and specific structures, GPCs can interact with a wide range of class of proteins, including morphogens, growth factors, cytokines, chemokines, ECM proteins and adhesion molecules [36]. Such interactions have been shown to play key roles in neoplastic growth and neovascularization. The exact mechanism behind the involvement of GPCs in malignant behavior is not known. A great challenge in this field is elucidating the individual contributions of the core proteins and the structurally diverse HS chains as signaling components in the dynamic and highly integrated tumor microenvironment. Detailed structural information regarding the interaction between the GPCs and different growth factors is limited because of lack of knowledge about the GPCs structures. So far, only structure of GPC1 has been solved by X-ray crystallography which reveals flexibility of the C-terminal region allowing freedom for GPC1 to orient and accommodate binding to receptors and other signaling molecules, presumably with the participation of the HS chains [42–44]. Reviews of the structural and functional features of HS proteoglycans and their signaling in the tumor environment point out their interaction with a variety of growth factors and involvement of complex downstream signaling pathways [1, 2]. As an attempt to reveal the mechanisms behind the mitogenic effects of GPC1 we performed IPA pathway analysis of the genes that were differentially expressed between *GPC1*-low and *GPC1*-high patients. The results reveal a cascade signaling involving the collagen family in the ECM, implicating pro-inflammatory mechanisms including interleukin and interferon signaling, finally converging to downstream known key mitogenic mechanisms such as TGF-β, p38 MAPK, AKT, PDGF-BB and WNT. These findings are in line with previous results uncovering the role of GPC1 in tumor microenvironment modulation and interaction with growth factors, receptors and major downstream pathways that lead to tumor growth, invasion and metastasis [for review see 1, 2, 9, 10, 32]. Network reconstruction data also revealed direct interaction between GPC1 and S100A2 which is directly involved in regulation of cell cycle and differentiation via its 2 EF-hand [33]. An interaction between GPC1 and H1-3 was also suggested by network reconstruction indicating involvement of GPC1 in gene transcription. To our knowledge this is the first time GPC1 has been connected to S100 and histone protein families. Of note, GPC1 and S100A2 have been previously reported together as candidate biomarkers associated with unfavorable prognosis in lung adenocarcinoma [45] without however relationship between the two molecules inferred. This finding offers ground for further research is to investigate physical and functional interactions. Molecular activity prediction analysis revealed the inhibition of TGFβ1 as a central process. Early studies in pancreatic cancers identify correlation between GPC1 expression and TGF-β [46, 47] suggesting the relationship between the two molecules. TGFβ1 was further connected with downstream inactivation of C-C motif chemokine receptor 2 involved in angiogenesis and activation of the nuclear receptor subfamily 4 group A member 1 (NR4A1) as well as the FAS receptor which is involved in apoptosis and inflammation. Taken together, these results highlight a putative mechanism where suppression of GPC1 leads to ECM-mediated inhibition of a number of complex and multifaceted mitogenic factors such as TGFβ and MAPK which in turn results in reduced signaling activation for malignant cell proliferation, angiogenesis and invasiveness.

This study was designed to increase the knowledge on the potential of GPCs and in particular GPC1 as a biomarker in cancer diagnosis and prognosis. It is plausible to measure circulating GPCs in serum, plasma or urine using a variety of methods including ELISA, urine cell sediments or exosome isolation [13, 24]. Further, detection and quantification of GPC1 by histopathological and immunohistochemical methods in tumor biopsies could be a new way to predict the biological outcome. The results of this investigation would also emphasize the potential of GPCs as novel tumor antigens, and open for GPC targeted immunotherapy. GPC targeted immunotherapy would be of high value, especially as we move into an era of precision and personalized cancer therapy.

## MATERIALS AND METHODS

### TCGA data preprocessing

Harmonized gene expression and clinical data from The Cancer Genome Atlas (TCGA) available at the NCI’s Genomic Data Commons (GDC) were downloaded using the R package TCGAbiolinks [48–50]. The data files were read and subjected to all further analysis using R, v. 4.0.6 [R Core Team, 2013]. Gene expression (HTSeq-Count) of GPC genes (GPC1-6) was obtained from 33 cancer types (Table 1). Gene counts were logarithmized (base 10) and standardized prior to survival analyses.

### Univariate survival analysis

The association between expression of any given GPC gene and overall survival was first tested in a univariate approach utilizing the R packages *survival*, v. 3.2-7 [51] and *survminer*, v. 0.4.8 [52]. Only primary tumor samples and patients with available vital status and survival/follow-up time were included. Each of the 6 GPC genes (*GPC1-6*) was tested individually against each cancer type. Initially, a continuous univariate Cox PH (proportional hazards) model was fitted for every gene to identify relationships between gene expression and survival. Subsequently, for every significant relationship between a gene and a cancer type, subjects were stratified based on gene expression level into “Low” (gene expression <25th percentile), “Med” (≥25th percentile and <75th percentile) and “High” (≥75th percentile), and a Kaplan-Meier univariate model was fitted. Kaplan-Meir curves were plotted using the function *ggsurvplot*.

### Multivariate survival analysis

Following the univariate survival analysis, a multivariate CoxPH survival analysis was performed for each cancer type testing survival against all GPC genes across the three gene expression strata. The function *coxph* was used to run the Cox regression. Forest plots illustrating the hazard ratios of each variable were generated using the function *ggforest*.

### Differential expression (DE) analysis

In this analysis we considered subjects from 10 cancer types where *GPC1* expression levels displayed significant results in the CoxPH univariate survival analysis. For each cancer type the HTSeq gene counts were obtained and subjects (samples) were stratified into two groups based on *GPC1* expression levels. Subjects where the *GPC1* counts were above the 75th percentile were labeled as “GPC1-high” while subjects with *GPC1* counts below the 25th percentile were labeled as “GPC1-low”. Differential expression analysis was performed for each cancer type using the DESeq2 package [53]. Pairwise Wald tests between GPC1-low vs. GPC1-high groups were performed and differentially expressed genes were filtered for Benjamini-Hochberg adjusted *p*-value < 0.05 and absolute log2 Fold Change > 0.58 (1.5-fold).

### Ingenuity pathway analysis (IPA)

Genes that were identified as differentially expressed (DE) between GPC1-low and GPC1-high patient groups (significance thresholds: adjusted *p*-value < 0.05 and |log2 foldchange| >0.58) in at least 5 of the studied TCGA cancer types (66 genes total) were subjected to IPA core analysis [54]. Canonical pathway enrichment analysis was performed using differentially expressed genes and the significance values (*p*-value of overlap) for the IPA Canonical Pathways were calculated by the right-tailed Fisher’s Exact Test, and the *p*-values were adjusted for multiple testing using the Benjamini-Hochberg correction. A ratio was calculated of the number of DE molecules associated with a given pathway divided by the total number of molecules in the reference set that map to the pathway. IPA also calculated for each pathway a z-score that predicted pathway activation if positive or inhibition if negative. The z-score is calculated by comparing the dataset fold changes under analysis with the canonical pathway patterns in the IPA Knowledge Base. Z-scores of ≥ 2 or ≤ −2 are considered significant, and no z-score annotation indicates either zero (or very close to zero) z-score or that the given pathway is ineligible for a prediction. Significant canonical pathway terms were filtered for BH adjusted *p*-value < 0.05. IPA Networks algorithm generated interaction networks of the input DE molecules, scoring the networks based on the count of network eligible molecules that they contained (molecules with known scientific evidence of directly or indirectly interacting with other molecules in the Ingenuity Knowledge Base). The score was based on the hypergeometric distribution and was calculated with the right-tailed Fisher’s Exact Test; the higher the score, the lower the probability of finding the observed number of the input dataset molecules in a given network by random chance.

### Cell culture

Urinary bladder carcinoma cells isolated from an 81 years old female patient (T24 cells), hepatocellular carcinoma cells isolated from a 15-year-old male patient (HepG2) and malignant glioma cells isolated from a male patient (U-87) were obtained from ATCC (cat# HTB-4; HB-8065 and ATCC, HTB-14 respectively; ATCC). Authentication and certificate of analysis was provided by ATCC. The cells were cultured according to instructions provided by ATCC. All cancer cells were routinely treated with mycoplasma removal agent for a week after thawing of frozen cells (cat# 3050044; MP Biomedicals).

### CRISPR/Cas9 targeting GPC1

The cells were transfected either with a pair of human GPC1 targeted CRISPR/Cas9 knockout plasmids (CRISPR/Cas9GPC1) (cat# sc-402002-NIC; Santa Cruz Biotechnology, Dallas, TX, USA) or a non-specific CRISPR/Cas9 control plasmid not targeting any known gene (CRISPRcontrol) (cat# sc-437281; Santa Cruz Biotechnology) according to the manufacturer’s instructions. Both plasmids encoded a GFP marker to indicate transfection. Successful transfections were determined by detection of GFP via fluorescence microscopy. Expression of GPC1 was assessed by immunofluorescence microscopy and further quantified by slot blotting of cell extracts. For each slot blot a total of 4-5 distinct samples were analyzed. The slot blots were further stripped and reprobed with β-tubulin antibody (cat# A-11126; Molecular probes) as loading control.

### Downregulation of *GPC1* expression by siRNA

Downregulation of *GPC1* expression by siRNA (siRNAGPC1) was performed as described before [55–57]. The vector pSilencer2.0-U6 (cat# 7209; Ambion Inc., Austin, TX, USA) containing sequence GCTGGTCTACTGTGCTCAC (corresponding to nucleotides 977–995 in human GPC1) followed by hairpin sequence TTCAAGAGA and then reversed complementary GPC1 sequence with an additional C in the 5′-end and a stretch of six Ts for RNA polymerase III termination followed by GGAA in the 3′-end was synthesized by Genscript Corp. A negative control vector comprising a scrambled sequence not targeting any known gene was also prepared (siRNAmock). Transfection was accomplished using FuGENE 6 Transfection Reagent (cat# E2691; Promega Biotech AB) following the instructions from the manufacturer. Silencing of GPC1 expression was verified by immunofluorescence microscopy and the level of silencing was quantified by slot blotting of cell extracts. For slot blots a total of 4–5 distinct samples were analyzed. The slot blots were further stripped and reprobed probed with β-tubulin antibody (cat# A-11126; Molecular probes) as loading control.

### Anti-GPC1 antibody

The polyclonal anti human GPC1 antibody has been described, validated and used many times before [57, 58]. A rabbit antiserum against human GPC1 was obtained after immunization with a 6-His tagged recombinant GPC1 core protein comprising the sequence Ile 54 to Pro 519. To generate the protein, human GPC1 cDNA was cleaved with BglII and StuI and ligated into the vector pQE32 (Qiagen) digested with BamHI/SmaI. The resulting plasmid was used to transform E. coli M15 bacteria. Protein expression was induced with IPTG (Gibco BRC). 6-His tagged protein was purified in guanidine HCl on a Ni^2+^-NTA-agarose column.

### Overexpression of GPC1 using ectopic expression of green fluorescent protein-tagged Gpc-1 (GFP-GPC1)

Overexpression of GPC1 has been described elsewhere [59]. To create a GFP-Gpc-1 vector, the Clontech vector pEGFP C1 was used. The sequence coding for the N-terminal signal peptide was amplified from cDNA by PCR. The PCR product was digested with AgeI/NheI and ligated into AgeI/NheI-digested pEGFP C1. A Kozak sequence was also introduced with the forward primer. The sequence coding for the core protein and C-terminal signal peptide was also amplified by PCR. The PCR product was digested with HindIII/EcoRI and ligated into HindIII/EcoRI-digested pEGFP C1. The start codon present in the sequence for enhanced GFP was disrupted by using site-directed mutagenesis. All mutations and constructs were verified by sequencing at Eurofins MWG Operon (Ebersberg, Germany). The cells were transiently transfected with the vector containing GFP-Gpc-1 for 72 h using Promegas standard protocol for transfection with FuGENE 6 Transfection Reagent (cat# E2691; Promega Biotech AB). The level of GPC1 expression was assessed by immunofluorescence microscopy and quantified by slot blotting of cell extracts. For slot blots a total of 4-5 distinct samples were analyzed. The slot blots were further stripped and reprobed with β-tubulin antibody (cat# A-11126; Molecular probes) as loading control.

### Immunofluorescence microscopy

Expression of GPC1 was examined by immunofluorescence microscopy as described previously [57, 59]. In detail, cells transfected with CRISPRcontrol, CRISPR/Cas9GPC1, siRNAGPC1, siRNAmock, or GFP-GPC1 were washed with PBS (137 mM NaCl, 2.7 mM KCl, 8 mM Na2HPO4, and 2 mM KH2PO4, pH 7.4) and fixed in acetone in order to retain cellular and subcellular structures. The fixed cells were first pre-coated with 10 % anti-rabbit total Ig and then exposed to polyclonal rabbit anti human GPC1 antibody (dilution 1:500) overnight. After extensive washings with PBS, the cells were treated with Alexa Fluor 594-tagged goat anti-rabbit IgG (cat# A-21208; Molecular Probes, dilution 1:500) for 4 h. To visualize nuclei, DNA staining was performed with 4′,6-diamidino-2-phenylindole (DAPI; Thermo Fisher Scientific; diluted to 300 μM). In the controls, the primary antibody was omitted. The fluorescent images were analyzed by using a Carl Zeiss AxioObserver inverted fluorescence microscope equipped with objective EC “Plan-Neofluar” 63 X/1.25 Oil M27 and AxioCam MRm Rev Camera. Identical exposure settings and times were used for all images. The fluorophores were excited in a sequential manner using multitrack acquisition to minimize channel cross-talk. During microscopy, the entire slides were scanned at 20× magnification and immunofluorescence images were retrieved at 60× or 100× magnifications.

### Slot blot assay

Cells (2 × 10^4^ cells) were extracted with radio-immunoprecipitation assay buffer (RIPA) (0.1% w/v SDS, 0.5% v/v Triton X-100, 0.5% w/v sodium deoxycholate in PBS) supplemented with cocktail of proteinase inhibitors cOmplete mini (cat# 11836153001; Roche) by shaking for 10 min at 4°C. Protein concentrations were determined with bicinchoninic acid assay using Pierce^™^ BCA Protein Assay Kit (cat# 23225; Thermo Fisher Scientific) according to manufacturer’s instructions. Samples were normalized in extraction buffer to protein concentrations of 1 mg/ml and equal amount of proteins were loaded on the PVDF membranes using slot blot. The PVDF membranes were incubated with anti-GPC1 antibody (1:500 dilution) for 5 h at room temperature or overnight at 4°C followed by extensive washing with PBS containing 0.5% Tween-20 and then treatment with horseradish peroxidase-conjugated anti-rabbit IgG (cat# 170-6515; Bio-Rad, Hercules, CA, USA; dilution 1:500). The membranes were further developed by chemiluminescence (cat# 35050; Pierce fast western blot kit) using Amersham ImageQuant 500 detector from Cytiva. Staining intensities were recorded by densitometry using GelAnalyzer 19.1 (http://www.gelanalyzer.com/). For loading control, the PVDF membranes were stripped in stripping buffer (1,5 w/v Glycin, 0.1 w/v SDS, 1 v/v Tween 20, pH 2.2) for 30 min at room temperature. The membranes were then incubated with β-tubulin antibody (cat# A-11126; Molecular probe,) followed by treatment with horseradish peroxidase-conjugated anti-mouse IgG (cat# 172-1011; Bio-Rad, Hercules, CA, USA; dilution 1:500). The level of GPC1 expression was adjusted according to β-tubulin levels when required. Negative controls omitting primary antibody showed no signal.

### Proliferation rate assay using crystal violet

The method has been described before [60]. Confluent cells were dissociated using TrypLETM (cat# 12604-021; Thermo Scientific) and seeded in 96-well microculture plates at plating density of 5000 cells/well. After 24 h of plating, the cells were left untreated or were treated with CRISPRcontrol, CRISPR/Cas9GPC1, siRNAmock, siRNAGPC1, GFP-GPC1 or anti-GPC1 polyclonal antibody (1:200). After 3–4 days the cells were fixed in 0.25% (v/v) glutaraldehyde in Hanks’ balanced salt solution for 30 min, and the cell density was measured by staining of the nuclei with 0.1% (v/v) crystal violet (cat# C6158; Sigma-Aldrich) for 30 min. The cells were then washed extensively with water and lysed in 1% (v/v) Triton X-100 for 4 h, followed by measurement of the amount of bound dye at A595 nm using Byonoy microplate reader (cat# ABSMHA01; Absorbance 96 compact ELISA reader, Byonoy). Cell proliferation rate was calculated using Byonoy Absorbance 96 software and MS Excel. Untreated cells and blanks containing only medium were included as controls. The relative cell number was calculated as % of untreated cells or controls (CRISPRcontrol or siRNAmock). The results are presented in graphs for experiments performed in duplicates, *n* = 5 in each experiment. The data points are shown as the means ± SE.

### Statistical analyses

The data points in the graphs are shown as the means ± SE, *n* = 5 in each experiment. For statistical analysis, two group comparisons were performed using unpaired two tailed student *t*-test and unequal variances data analysis. Error probabilities of *P* ≤ 0.05 were considered statistically significant. Indication of *P*-value summaries: ns *P* > 0.05, ^*^ *P* ≤ 0.05, ^**^ *P* ≤ 0.01, ^***^ *P* ≤ 0.001, ^****^ *P* ≤ 0.0001.

## SUPPLEMENTARY MATERIALS


