# Supplementary material for: Attenuation of cancer proliferation by suppression of glypican-1 and its pleiotropic effects in neoplastic behavior
Source: Oncotarget. 2023 Mar 21;14:219–35. doi: 10.18632/oncotarget.28388 (PMC10030152; doi:10.18632/oncotarget.28388)
Supplement: Supplementary file 1 [file oncotarget-14-28388-s001.pdf]

Attenuation of cancer proliferation by suppression of glypican-1 and its pleiotropic effects in neoplastic behavior

SUPPLEMENTARY MATERIALS

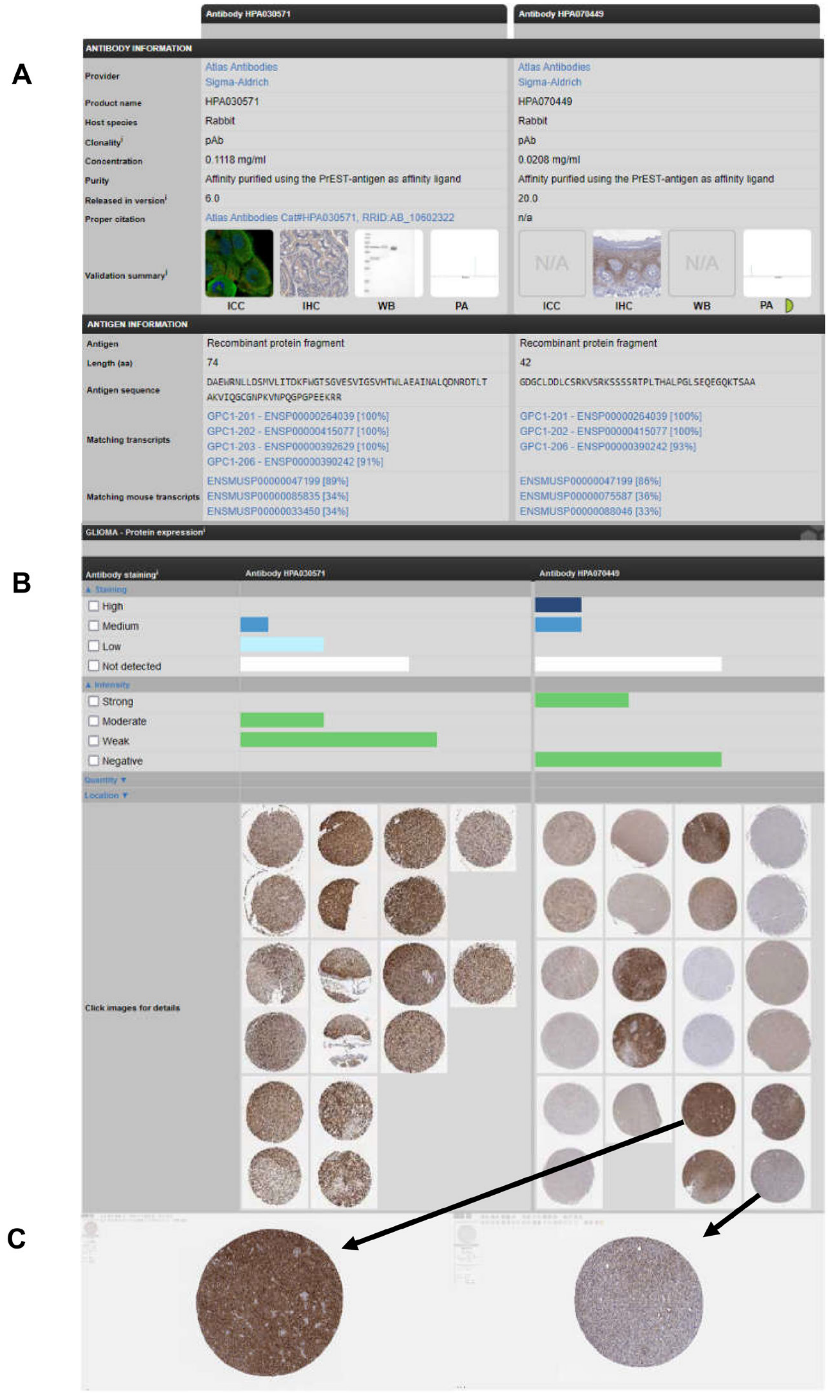

Supplementary Figure 1: Data from The human protein atlas shows difference in detection levels for two different GPC1 antibodies in biopsy samples from malignant glioma patients [1]. (**A**) Antibody data and antigen information for two different GPC1 antibodies (https://www.proteinatlas.org/ENSG00000063660-GPC1/summary/antibody). (**B**) Variations in detection levels for the two different GPC1 antibodies in biopsies from the same cancer type i.e., malignant glioma cancer. (**C**) Variations in detection levels for the same antibody in biopsies from the same cancer type (malignant glioma cancer).

Supplementary Table 1: List of significant (adjusted $p < 0.05$) canonical pathways enrichment results

Ingenuity Canonical Pathways	-log (*p*-value)	zScore	Ratio	GeneNames	*P*-value	Adj. *p*-value
Wound Healing Signaling Pathway	9.12	−2.5298221	0.0397	COL11A1,COL1A1,COL1A2,COL22A1,COL5A1,COL6A1,COL6A2,COL8	7.5858E-10	4.4778E-08
GP6 Signaling Pathway	8.94	−2.1213203	0.063	COL11A1,COL1A1,COL1A2,COL22A1,COL5A1,COL6A1,COL6A2,COL8	1.1482E-09	4.4778E-08
Hepatic Fibrosis/Hepatic Stellate Cell Activation	7.49		0.0412	COL11A1,COL1A1,COL1A2,COL22A1,COL5A1,COL6A1,COL6A2,COL8	3.2359E-08	8.4134E-07
Neutrophil Extracellular Trap Signaling Pathway	7.08	2.52982213	0.0242	COL11A1,COL1A1,COL1A2,COL22A1,COL5A1,COL6A1,COL6A2,COL8	8.3176E-08	1.2976E-06
Role Of Osteoclasts In Rheumatoid Arthritis Signaling Pathway	7.08	−1.6666667	0.0291	COL11A1,COL1A1,COL1A2,COL22A1,COL5A1,COL6A1,COL6A2,COL8	8.3176E-08	1.2976E-06
Pulmonary Fibrosis Idiopathic Signaling Pathway	6.88	−2.3333333	0.0276	COL11A1,COL1A1,COL1A2,COL22A1,COL5A1,COL6A1,COL6A2,COL8	1.3183E-07	1.7137E-06
Pathogen Induced Cytokine Storm Signaling Pathway	5.36	−2.1213203	0.0216	COL11A1,COL1A1,COL1A2,COL22A1,COL5A1,COL6A1,COL6A2,COL8	4.3652E-06	4.864E-05
IL-4 Signaling	3.99	−2.1213203	0.0139	COL11A1,COL1A1,COL1A2,COL22A1,COL5A1,COL6A1,COL6A2,COL8	0.00010233	0.00099771
Apelin Liver Signaling Pathway	2.67		0.0741	COL1A1,COL1A2	0.00213796	0.018529
Intrinsic Prothrombin Activation Pathway	2.29		0.0476	COL1A1,COL1A2	0.00512861	0.04000319

SUPPLEMENTARY REFERENCE

1. Uhlen M, Zhang C, Lee S, Sjöstedt E, Fagerberg L, Bidkhori G, Benfeitas R, Arif M, Liu Z, Edfors F, Sanli K, von Feilitzen K, Oksvold P, et al. A pathology atlas of the human cancer transcriptome. Science. 2017; 357:eaan2507. https://doi.org/10.1126/science.aan2507. [PubMed]
